# Interpretation of common endocrine laboratory tests: technical pitfalls, their mechanisms and practical considerations

**DOI:** 10.1186/s40842-019-0086-7

**Published:** 2019-07-24

**Authors:** Raad A. Haddad, Donald Giacherio, Ariel L. Barkan

**Affiliations:** 1 Division of Metabolism, Endocrinology and Diabetes, Department of Internal Medicine, University of Michigan Medical Center, 24 Frank Lloyd Wright, G-1500, Ann Arbor, MI 48106 USA; 2 Department of Pathology, University of Michigan Medical Center, Ann Arbor, MI USA; 3 Department of Neurosurgery, University of Michigan Medical Center, Ann Arbor, MI USA

**Keywords:** Immunoassays, Interpretation, Hormones, Pitfalls

## Abstract

Pitfalls in hormonal assays are commonly seen in clinical practice and may lead to erroneous clinical impressions and treatments. In this article, we address common laboratory pitfalls encountered during evaluation of patients with real or presumed endocrine disorders including high dose hook effect and falsely normal prolactin in cases of macroprolactinomas, macroprolactinemia and falsely elevated prolactin, macrothyrotropinemia and falsely elevated TSH, heterophile antibodies leading to false elevation of hormonal concentration, biotin interference with different hormonal assays, cross-reactivity of steroid hormones immunoassays, and others. We describe the mechanisms of such laboratory pitfalls, review clinical scenarios in which they might occur, and discuss the ways to resolve such conundrums. The aim of this article is to present a learning material for the endocrine trainees and practitioners.

## Background

Accurate hormonal assays play a significant role in the practice of endocrinology. A few decades ago, development of the radioimmunoassay (RIA) was awarded the Nobel prize as a revolutionary tool for measuring peptide hormones [1]. This was followed by measurement of the non-immunogenic steroid hormones [1–3]. Despite advances in laboratory techniques in the last few decades, pitfalls in endocrine testing can commonly happen distorting the clinical picture.

Various laboratory methods are used to assess endocrine problems including immunoassays and more recently, mass spectrometry. Immunoassays remain the most commonly used method to evaluate hormonal disorders [4]. They can be mainly divided into two groups: competitive and noncompetitive immunoassays.

In a two-step competitive immunoassay, antibodies to human hormone are generated in an animal of a certain species (rabbit, goat, guinea pig, etc.), this polyclonal first antibody is added to a sample of a patient’s serum or plasma together with a known amount of a radiolabeled hormone of interest that competes for binding to the first antibody with endogenous hormone. After incubation, the bound fraction is precipitated by a second antibody that is generated against the immunoglobulin G (IgG) belonging to the species in which the first antibody was produced, the supernatant is discarded, and the radioactivity of a pellet containing bound both labeled and endogenous hormones is measured. Understandably, the more endogenous hormone is contained in the sample, the less labeled hormone will be bound (this is the essence of competition). Thus, the weaker the signal, the more of the endogenous hormone was present in the sample and vice versa.

**Fig. 1:**
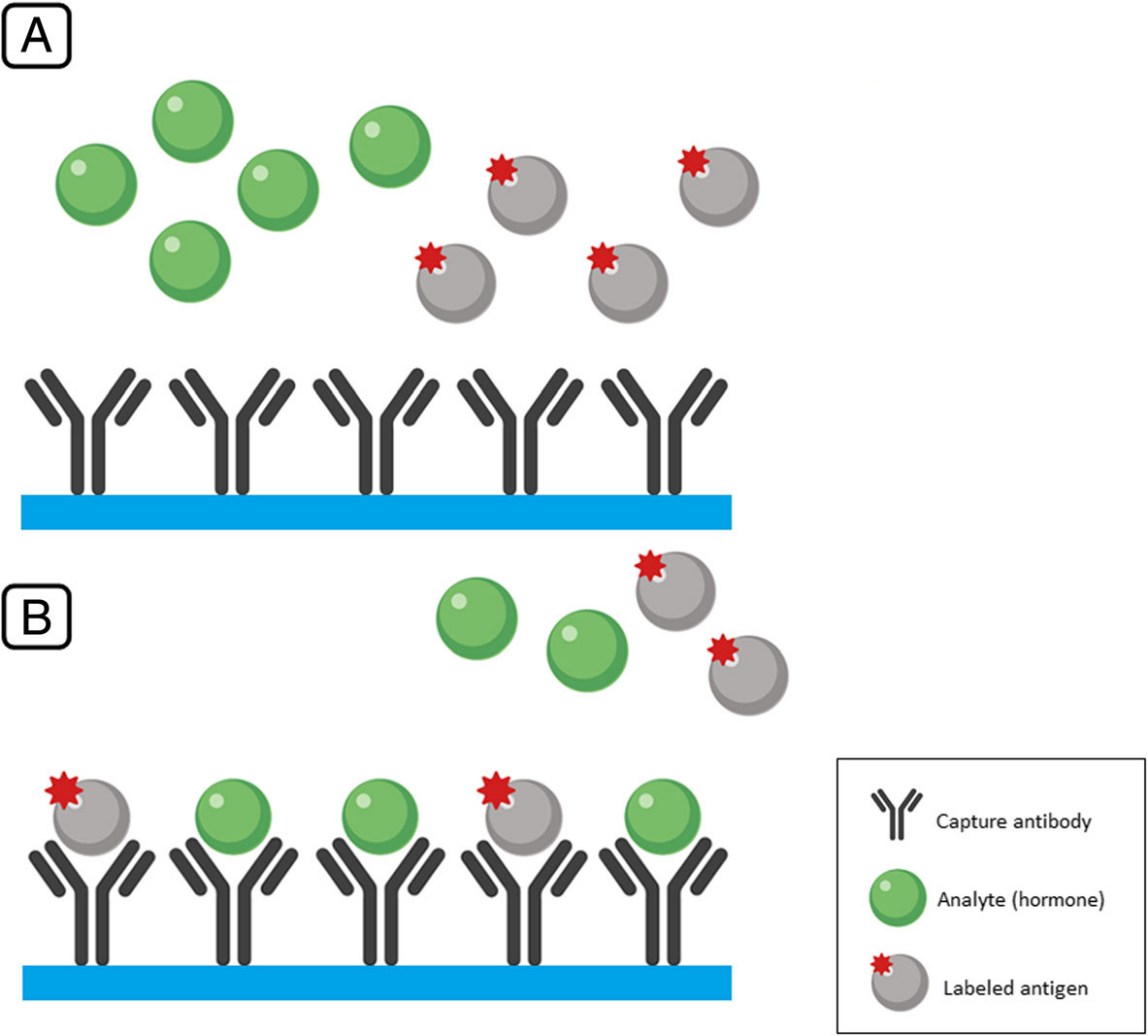
Illustration of single step competitive immunoassay. **a** In single step competitive immunoassay, capture antibodies are anchored to a solid phase in the testing tube. A certain concentration of labeled analyte (pre-prepared labeled antigen) is added to the testing tube together with the blood sample containing the studied hormone. **b** Both the studied analyte (the hormone) and the labeled analyte compete for the binding sites of the capture antibodies. After discarding the supernatant with both unbound endogenous and labeled analytes, the remaining signal of the bound labeled analyte is measured. The higher the concentration of the hormone (illustrated in green), the less labeled analyte will be bound, and thus, the less signal will be measured. In other words, the weaker the signal, the more of the endogenous hormone is present in the sample and vice versa (i.e. the signal strength is inversely proportional to the hormone concentration)

In clinical laboratories today, two step radioimmunoassay as described above has largely been replaced by non-isotopic single step competitive immunoassay. The antibody is immobilized on a solid surface, and a competition is set up by adding patient sample and a known concentration of labeled analyte. Chemiluminescent labels have become the dominant method for these assays (Fig. 1).

**Fig. 2:**
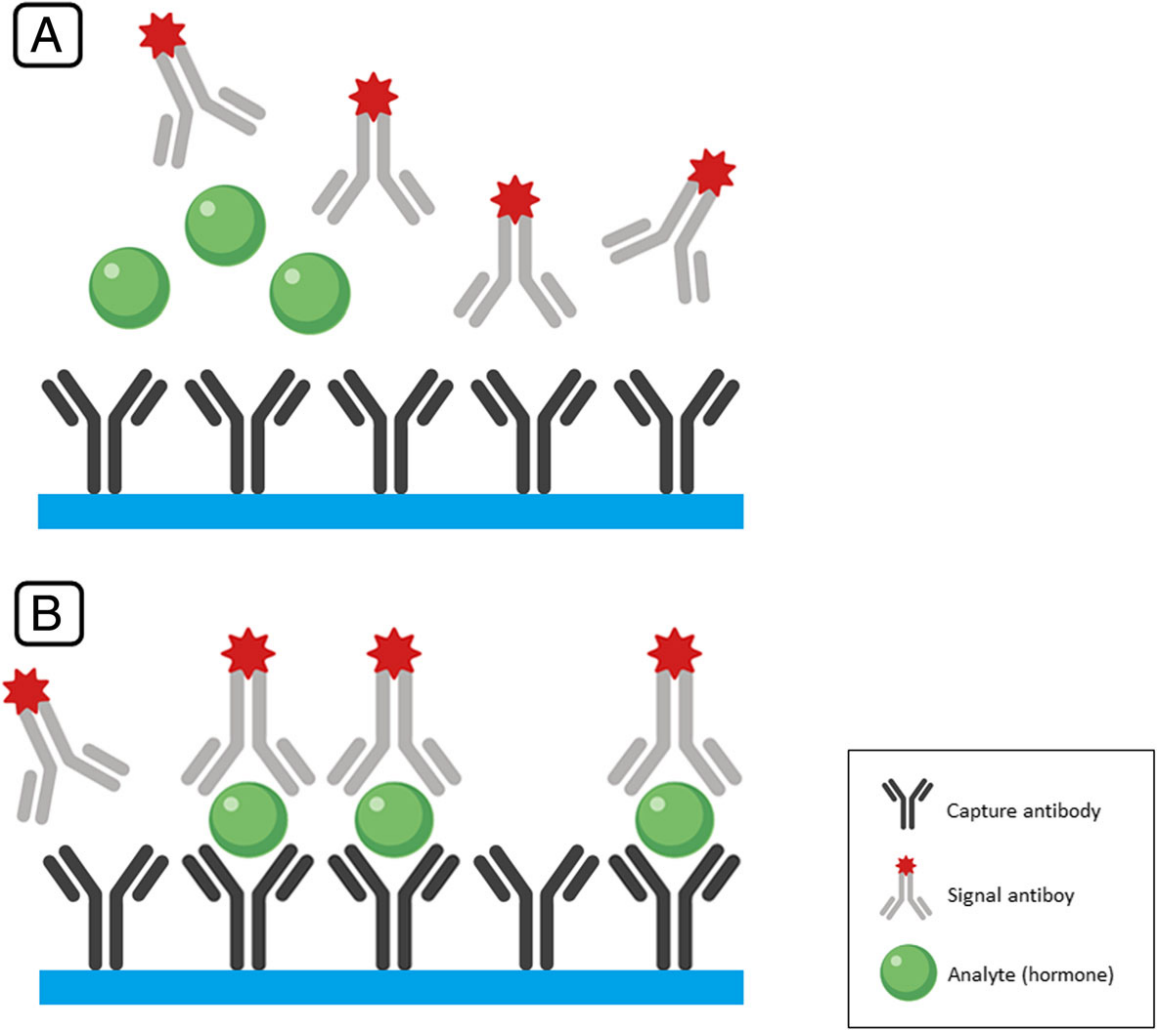
Illustration of non-competitive “sandwich” immunoassay. **a** In non-competitive “sandwich” immunoassay, capture antibodies are anchored to a solid phase in the testing tube. Then, the sample containing the studied hormone as well as the signal antibodies are added. **b** The hormone binds to the capture antibody from one end and to the signal antibody from the other end, forming antibody-hormone-antibody “sandwich”. The unbound signal antibodies is then washed out and the signal from the remaining “sandwiches” is measured. The higher the concentration of the hormone (illustrated in green), the more “sandwiches” will be formed, and thus, the higher signal will be measured. In other words, the stronger the signal, the more of the endogenous hormone is present in the sample and vice versa (i.e. the signal is directly proportional to the hormone concentration)

Noncompetitive assays employ two antibodies, but the mechanism of the assay is different. One monoclonal antibody is firmly attached to a solid surface of the test tube (the wall, plastic or glass ball, magnetic particles, etc.). It binds with the hormone in question and is called capture antibody. Another monoclonal antibody, labeled by various techniques (radioactivity, luminophore, fluorescent tag, etc.) is generated to a different epitope of the hormone in question and is called the signal antibody. It binds to the free epitope of the hormone in question already bound to the capture antibody and results in a formation of a so-called “sandwich”, i.e. a complex consisting of a capture antibody, hormone and signal antibody that is firmly attached to a solid surface. When the supernatant is discarded and the tube is gently washed, only the sandwich remains within it and the signal generated by the signal antibody is measured (Fig. 2). Understandably, the stronger the signal, the more hormone was contained in the serum sample and vice versa.

Limitations of these assays can be caused by many factors including pre-analytical sampling conditions, specificity of reagents used in the analysis, the presence of interfering or cross-reacting substances, and the presence of antibodies against either the reagents or the analyte. They can provide erroneous results leading the physician to pursue a wrong diagnosis and administer wrong treatment. When ordering a test, many factors need to be taken into consideration, including clinical suspicion of a problem, co-administration of various medications and reliability of the requested test. In this review article, we will highlight some pitfalls of commonly ordered endocrine tests that are important to consider for proper interpretation of a laboratory report. All pitfalls described here were encountered in our clinical practice in the Pituitary and Neuroendocrine Center at the University of Michigan, either during de-novo evaluation of a patient or, more often, during re-evaluation of patients who are erroneously diagnosed due to phantoms in the immunoassay.

### The high dose hook effect

**Fig. 3:**
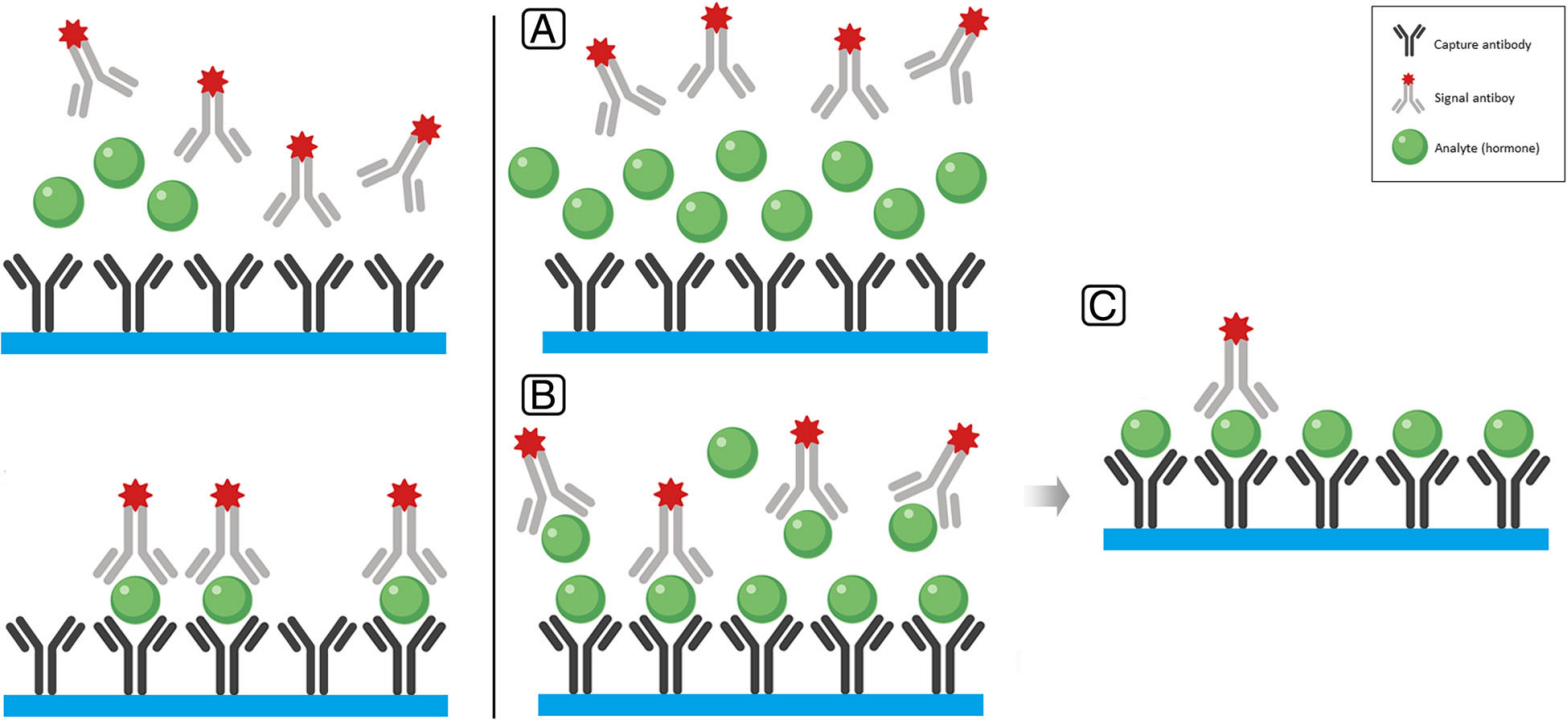
Illustration of the high dose hook effect. The left panel illustrates the non-competitive “sandwich” immunoassay with normal (or elevated within the tolerance of the assay kit) hormone concentration (see Fig. 2). The right panel illustrates the mechanism of the hook effect with exceedingly high hormone concentration. **a** The sample that contains remarkably elevated hormone concentration is added to the test tube which contains both capture and signal antibodies. **b** The studied hormone overwhelmingly saturates both the capture and signal antibodies preventing the formation of the “sandwiches”. **c** After the washout phase, only a few “sandwiches” will be left producing a low signal

This phenomenon happens with the use of immunoassays to measure a certain hormone to be measured (analyte), in particular when using the two-site monoclonal “sandwich” assay [5]. Both serum sample and the signal antibody are added to the test tube simultaneously. As explained earlier, one epitope of the hormone binds to the capture antibody, while the other epitope of the hormone binds to the signal antibody, forming an antibody-hormone-antibody “sandwich” firmly attached to the solid surface. After the liquid component is discarded, the solid surface attached “sandwiches” elicit a signal that is directly proportional to the hormone concentration in the sample [6]. However, when the hormone concentration is exceedingly high or the amount of antibodies put by the manufacturer in the kit is low, the hormone saturates both the capture and signal antibodies preventing formation of the “sandwich”. As a result, after the liquid component is discarded, there will be only limited numbers of “sandwiches” attached to the solid surface and the detected signal will indicate low or only mildly elevated analyte concentration (Fig. 3). The shape of the binding curve gave the name “hook effect” to the phenomenon; with gradually-increasing analyte concentrations in the sample, the binding curve goes up, but at some critical point exceeding the capacity of the assay components, it starts “hooking down”. Thus, in cases of prolactinoma, for example, with exceedingly high prolactin levels, the false report of only mildly elevated prolactin may force the physician to make an erroneous diagnosis of a non-functioning pituitary tumor and subject the patient to unnecessary surgery (with potential complications) instead of prescribing dopamine agonists [7].

How to prevent a potential hook effect phenomenon from making the clinical picture confusing? The presence of large deposits of neoplastic tissue is a necessary clinical clue. Understandably, only extremely high concentrations of analyte can cause the hook effect to occur. Since the magnitude of analyte concentration in the serum is as a rule proportionate to the size of a secreting tumor, only pituitary macroprolactinomas (usually above 4 cm) and malignant and widely metastatic tumors are likely to present this problem. The published list of other tests susceptible to this hook effect includes beta human chorionic gonadotropin (B-HCG) in patients with choriocarcinoma, thyroglobulin in thyroid cancer, and prostate-specific antigen in patient with metastatic prostate cancer [8–10]. In all the above situations, glossed over “normal” or “modestly elevated” serum markers can miss the diagnosis and withhold the needed therapy. The hook effect has not been described in cases of somatotropinomas (acromegaly) or corticotropinomas (Cushing disease) likely because these tumors are rarely big enough to produce astronomical hormone concentrations. Manufacturers of laboratory kits are aware of this problem and current kits often contain high enough concentrations of antibodies. For example, a prolactin assay employed in our hospital laboratory (ADVIA Centaur XP chemiluminometric assay by Siemens Diagnostics) is protected against the hook effect up to serum prolactin concentrations of 60,000 ng/mL. However, in one of our patients with a 9 cm skull base tumor, serum prolactin was 280,000 ng/mL [6]. Thus, a physician’s vigilance is still needed. Any large (above 4 cm) pituitary mass suspected of being an adenoma needs to be evaluated for the presence of hook effect.

One way of establishing true hormone concentration is to perform a sample dilution 1:100 or even more prior to adding it to the assay tube and then multiplying the results by the dilution factor. Another way is to incubate the serum sample with the capture antibody first, discard the supernatant containing the non-bound analyte (hormone) and only then add the signal antibody. Unfortunately, this latter technique is rarely an option with United States Food and Drug Administration (FDA) approved automatic immunoassays.

### Macroprolactinemia

Prolactin is a human peptide hormone that is synthesized in the anterior pituitary gland in its pre-hormonal form. The pro-hormone undergoes cleavage and gets converted to the monomeric prolactin that weighs ~ 23 kDa. It is the most abundant form in the serum and known to be biologically and immunologically active [11]. This monomeric form is one of three forms of prolactin based on different molecular sizes; the two other forms include dimeric prolactin with a molecular weight of ~ 50 kDa and macroprolactin with a molecular weight of more than 100 kDa [12]. Macroprolactin, in fact, is a large prolactin-IgG antibody complex and is known to be biologically inactive [12, 13]. In normal subjects, the proportions of monomeric, dimeric, and macroprolactin were reportedly 85.8 ± 2.3, 9.1 ± 0.9, and 5.1% ± 1.7%, respectively [14].

Macroprolactinemia occurs when the high molecular weight macroprolactin predominates in the serum [15]. Macroprolactin is coupled with IgG forming a complex that has low receptor affinity and thus is biologically inactive [16]. The pathogenesis of macroprolactinemia remains unclear, Hattori et al. suggested that post-translational modifications can induce immunogenicity and formation of anti-prolactin autoantibodies resulting in high deposits of macroprolactin [17].

Macroprolactin interferes with most immunoassays used for prolactin level measurement leading to falsely elevated prolactin level. At the same time, macroprolactin does not yield to negative hypothalamic feedback, and thus, a true hyperprolactinemia can be further increased [18]. This can lead to misdiagnosis, mismanagement, and unnecessary consumption of medical resources. Among other many causes of hyperprolactinemia, macroprolactinemia should be considered especially when the clinical scenario lacks the clinical and the radiological evidence of hyperprolactinemia. In some studies, the prevalence of macroprolactinemia was as high as 26% of patients with apparent hyperprolactinemia [19]. It is important to note that patients with systemic lupus erythematosus (SLE) are especially prone to developing macroprolactinemia due to the presence of anti-prolactin antibodies; an estimated one-third of patients with SLE and hyperprolactinemia were found to have macroprolactinemia [20].

The gel filtration chromatography (GFC) remains the gold standard procedure to differentiate between different molecular forms of prolactin. However, this method is time and labor intensive [21]. Polyethylene glycol (PEG) precipitation has become a more commonly used screening method, given its ease and cost effectiveness. Adding PEG precipitates macroprolactin, leaving monomeric prolactin in the supernatant. Macroprolactinemia is usually suspected when PEG-precipitable prolactin exceeds 60% of the total prolactin, in other words, when the monomeric prolactin in the supernatant is less than 40% [22].

### Macrothyrotropinemia (macro-TSH)

Thyroid stimulating hormone (TSH) is a commonly measured hormone in clinical practice. Similar to macroprolactin, anti-TSH autoantibodies form an antigen-antibody complex consistent of TSH and anti-TSH, forming what is known as macro-TSH with low bioactivity. This complex may affect some commercially available TSH assays, resulting in falsely elevated TSH levels [23].

Patients are usually euthyroid with normal thyroxine (T4) and triiodothyronine (T3) levels. Macro-TSH may mimic primary hypothyroidism and can be very challenging to suspect. This phenomenon is less prevalent compared to macroprolactinemia; in a study done by Mills et al., the prevalence of elevated TSH due to macro-TSH was found to be 0.6% [24].

In contrast to macroprolactinemia, PEG-precipitation method was not found reliable; nonspecific precipitation ratios were much higher for TSH than prolactin [25]. This finding concluded that macro-TSH should be suspected when PEG-precipitable TSH exceeds 90%, especially, when serum TSH is greater than 10 mU/L. Confirmation can be conducted by using gel filtration chromatography.

### Heterophile antibodies

**Fig. 4:**
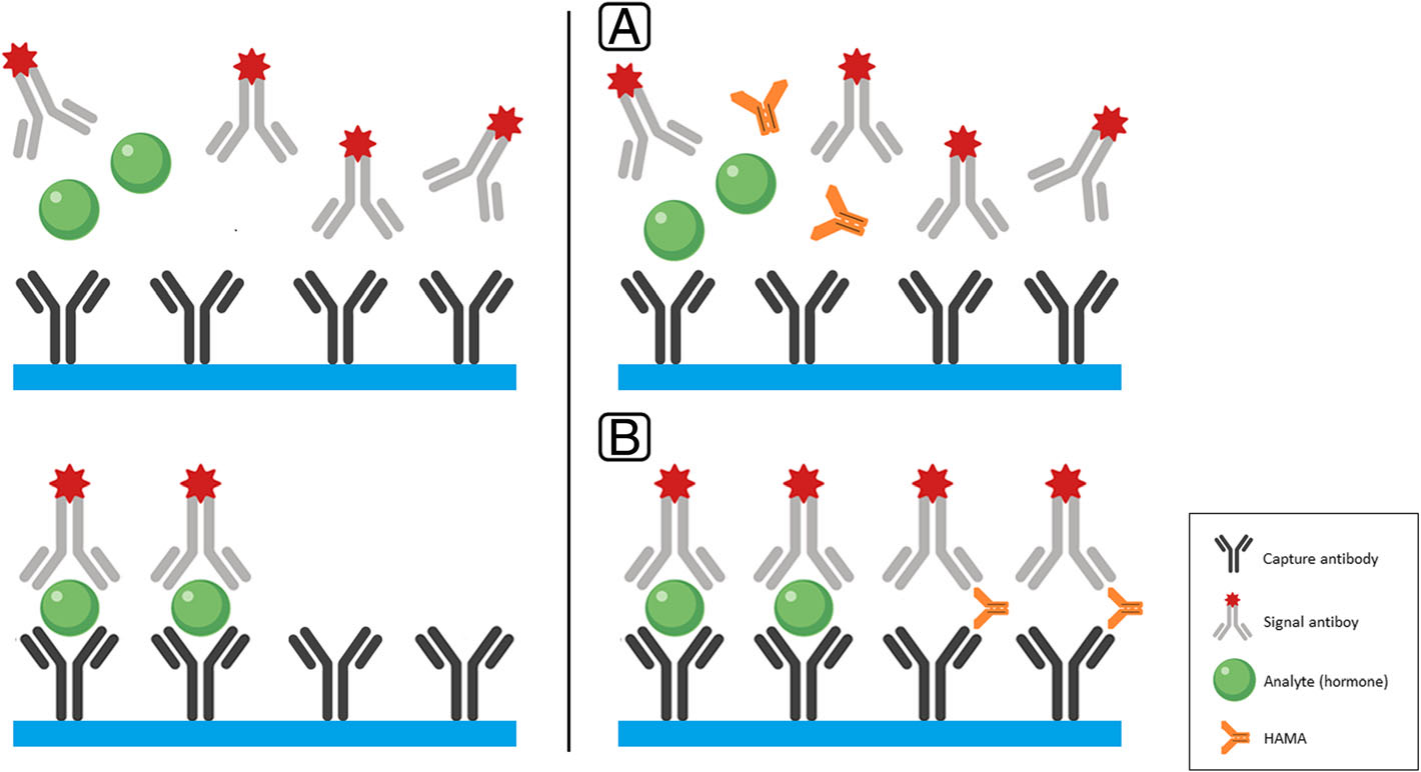
Illustration of human anti-mouse antibodies (HAMA) interference with immunoassay. The left panel illustrates the non-competitive “sandwich” immunoassay without the presence of HAMA in the sample (see Fig. 2). The right panel illustrates the mechanism of the HAMA interference with immunoassay. **a** The sample containing the studied hormone and the HAMA is added to the test tube which contains both capture and signal antibodies. **b** In addition to the correct formation of “sandwiches” (capture antibody- hormone-signal antibody), the HAMA forms a bridge between the capture antibody and the signal antibody forming antibody-HAMA-antibody “sandwiches”. As a result, more signal will be measured, and thus, false elevation of the studied hormone will be reported

Heterophile antibodies (HAB) are antibodies that are formed due to exposure to external antigens. Animal antigens can be involved in forming what is called human anti-animal antibodies. A common antibody that falls in this category is human anti-mouse antibodies (HAMA) [26]. They are endogenous antibodies that form against murine monoclonal immunoglobulin [27]. In immunometric “sandwich” assays, HAMA can form a bridge between the capture and signal antibodies (Fig. 4), forming more “sandwiches”, leading to a falsely elevated signal of the studied analyte [28].

The prevalence of HAB wildly varied in different studies. In a study by Koshida et al., overall, the prevalence of HAMAs in randomly collected samples was 11.7% [29]. These antibodies can interfere with a myriad of immunoassays including thyroid stimulating hormone, human chorionic gonadotropin (hCG), a-fetoproteins (AFP), cancer antigen 125 (CA-125), creatine kinase-muscle and brain (CK-MB) isozyme, and troponin among others [29, 30]. It is noteworthy that rheumatoid factor is an endogenous antibody that can mimic HAB and interfere with such immunoassays [31, 32].

An unexpected elevation of a hormone raises the suspicion of such issue. Subclinical hypothyroidism is commonly seen in endocrinology practice; in the presence of HAMA, TSH can be falsely elevated. In such case, a hint for the presence of these antibodies is failure of TSH normalization despite escalating doses of levothyroxine to the thyrotoxic range [33]. Patients who interact with animals, such as veterinarians or technicians studying mice, or just people living in mice-infested houses, can add a clue to the clinical picture.

When interference is suspected, several maneuvers can be undertaken to overcome such issue. Samples can often be retested with a different immunoassay or methodology since a number of commercially available assays has been developed to minimize the effects of HAMA [34]. Analyzing the sample with heterophile blocking reagent or diluting the sample and testing for linearity can also be done [35]. Measuring HAB (or HAMA) may identify patients who had exposure to a certain immunogen, however, this method has limited value and cannot prove HAB as the cause of an elevated result [30]. Communication with the laboratory performing the assay can be helpful in this situation.

### Biotin interference with hormonal assays

Biotin, or vitamin B7, is a water-soluble vitamin that is involved in many enzymatic activities that regulate metabolism of fat, carbohydrates and amino acids [36]. Biotin is available in many plant and animal based sources of food. Despite the fact that biotin deficiency is rare, it is available in most of over the counter multivitamins preparations. Biotin supplementation has been increasingly used as an enhancer of skin, nails and hair health, despite the lack of evidence that supports that [37].

Biotin-streptavidin detection method is commonly used in many immunoassays [38]. Biotinylated antibodies (capture antibodies) bind strongly to streptavidin that anchors those antibodies to the solid phase of the assay. High biotin concentration in the serum interferes with this bond and alters the expected results [39]. In non-competitive “sandwich” immunoassays, the studied analyte (or hormone) is sandwiched between the capture and signal antibodies. In the presence of high biotin concentration in the specimen, biotin saturates streptavidin binding sites which alters the adherence between the capture (biotinylated) antibodies and streptavidin, leading to a low signal after the wash off phase (i.e. falsely low concentration of the analyte). In contrast, as in competitive assay, the studied analyte competes with the labeled analyte to bind to the specific antibodies. That means, the higher the concentration of the endogenous analyte, the less labeled hormone will bind to the antibody. In other words, the remaining signal after the wash off phase is inversely proportional to the analyte concentration. Thus, having high concentration of biotin in the specimen leads to lower signal of the labeled analyte, which maybe interpreted as falsely high concentration of the studied analyte in competitive immunoassays [40, 41].

The magnitude of biotin interference varies based on the concentration of biotin in the specimen, results may be distorted to be either falsely high or falsely low depending on the format of the assay used [42]. A broad spectrum of tests beyond hormonal assays can be affected by the presence of excess biotin in the serum, most commonly, thyroid function test; especially in the presence of a discrepancy between the TSH and free T4 levels. It is important to verify biotin use in patients, especially, if they present with non-matching clinical and biochemical picture. Biotin is excreted by the kidney with a half life between 8 and 16 h [43]. A simple way to overcome such interference is to stop biotin use for a few days before repeating the test. Another way is to use an alternative assay that does not depend on biotin-streptavidin technology. Not all immunoassay manufacturers use biotin-streptavidin coupling, so communication with the laboratory for guidance when interference is suspected is important. Some manufacturers incorporate reagents to eliminate the effect of exogenous biotin on the assay [44].

### Cross-reactivity of steroid hormones immunoassays

Immunoassays are widely used to measure steroid hormones in different body fluids including serum, saliva and urine. A major pitfall of using immunoassays, especially competitive immunoassays, in measuring steroid hormones is cross-reactivity with other structurally similar molecules or compounds that can be either endogenous or exogenous in source [35]. This compromise can lead to inaccurate values of the measured hormone leading to altered clinical impression.

In certain clinical situations, such as congenital adrenal hyperplasia (CAH), adrenal steroid intermediaries, 11-deoxycortisol and 17-hydroxyprogesterone for example, may rise in the serum and interfere with cortisol immunoassays [45]. In a study by Monaghan el al, exogenous inhibition of steroidogenesis using metyrapone (inhibiting 11β-hydroxylase) in patients with Cushing’s syndrome resulted in false elevation of serum cortisol due to the accumulation of 11-deoxycortisol [46].

A situation that we have encountered several times in our clinic is a referral of a patient with the diagnosis of Cushing’s syndrome; the patient is treated with prednisone for some reason and often is, indeed, clinically cushingoid. Moreover, plasma and urinary cortisol levels are high (or, at least “not suppressed”). In combination with undetectable adrenocorticotropic hormone (ACTH) levels, that often leads to falsely suspected endogenous Cushing’s syndrome and almost always fruitless search for an adrenal tumor. The commonly overlooked pitfall is that most cortisol immunoassays have cross-reactivity to prednisolone, the active form of prednisone, ranging from modest to significant. Thus, prednisolone cross-reacts in the cortisol assay and “elevated cortisol” reported by the laboratory is in fact predictably-present prednisolone.

Other steroid hormones assays, such as testosterone and estradiol, are also subject to cross-reactivity if structurally similar compounds are present in the serum. When suspected, liquid chromatography-tandem mass spectrometry (LC-MS/MS) has became a well-known and more available technique providing more accurate measurement of the targeted steroid hormones to overcome such quandary [47].

### Dexamethasone suppression test and cytochrome P450 3A4 (CYP3A4)

The overnight one milligram dexamethasone suppression test (DST) is commonly used to screen for Cushing’s syndrome [48]. Failure to suppress morning cortisol level below 1.8 mcg/dL raises such suspicion [49]. Serum dexamethasone has to reach certain level to be able to suppress the hypothalamic-pituitary-adrenal (HPA) axis, and thus, cortisol secretion. Certain conditions can impede cortisol suppression, including decreased dexamethasone absorption, enhanced dexamethasone metabolism and clearance, or in cases of pseudo-Cushing, such as psychiatric illness, obesity, alcoholism, and others [50, 51].

Dexamethasone is extensively metabolized via CYP3A4, in human gut and liver mainly into 6-hydroxydexamethasone and other metabolites [52]. Induction of CYP3A4 may result in fast dexamethasone metabolism, incomplete HPA axis suppression, and thus, false positive DST [53]. Various medications enhance CYP3A4 activity and lower serum dexamethasone levels such as phenobarbital, phenytoin, carbamazepine, primidone, mitotane, enzalutamide, apalutamide, rifampin, pioglitazone, St John’s wort and other medications [54–59]. It is noteworthy to mention that medications that increase cortisol binding globulin (CBG), like estrogen, may also lead to falsely positive DST by increasing the total cortisol level [60].

Before evaluation for possible Cushing’s via DST, it is essential to review patient’s comorbidities and medications that might lead to false positive test. It is also important to measure dexamethasone concentration in the serum at the time of measuring morning cortisol after suppression, this can provide an idea whether the desired dexamethasone concentration was actually achieved. If one milligram DST is doubtful, other screening tests can be pursued, such as twenty-four-hour urinary free cortisol (UFC), late-night salivary cortisol, late-night serum cortisol (if feasible), or two-day two milligram DST [50, 59].

### Analog methods for free testosterone and thyroxine

These hormones are frequently measured in clinical practice. However, one must remember that significant proportions of their measured total concentrations circulate in bound forms, being attached to their specific binding proteins sex hormone binding globulin (SHBG) and thyroxine-binding globulin (TBG) respectively, and therefore are biologically inactive. Some proportions of them are weakly bound to albumin, and dissociate from it easily; thus they do exert biological effects. Any alterations in the concentrations of SHBG and/or TBG will falsely increase or decrease total concentrations of their corresponding hormones and non-specific binding of some drugs may also do the same. Equilibrium dialysis is the current gold standard for measuring free hormone concentrations, but is very time and effort consuming. For that reason, the so-called “analog methods” have been developed; they are based on the competition of radiolabeled analogs of the above hormones with (allegedly) their free fractions. In reality, however, free testosterone concentrations by analog methods are linked not to the true free, but to the total testosterone [61]. For that reason the Endocrine Society did not endorse the universal use of analog free testosterone assays in clinical practice [62]. The so-called “bio-available” testosterone assay is currently widely used in clinical laboratories and gives information on the sum of free and albumin bound testosterone. It correlates sufficiently well with free testosterone measurements by equilibrium dialysis and is a useful index of biological changes [62].

Free thyroxine measurement by analog methods is relatively robust and is widely used. However, it is still a subject to erroneous results by some platforms due to the presence of familial dysalbuminemic hyperthyroxinemia, anti-rutenium interference, thyroid hormone autoantibodies, etc. [63].

### Unknown interfering substances

Occasionally, a patient may present with very confusing hormonal data that are often incompatible with the clinical picture and all the above mentioned reasons for that prove negative. In these infrequent cases, careful clinical examination, understanding of normal physiology of hormone synthesis, secretion and metabolism remain the only way to find an appropriate test(s) that suggest the presence of an unknown interfering substance [64]. Fortunately, such situations, although frustrating, are extremely rare.

## Conclusions

Laboratory diagnosis of endocrine diseases is an indispensable tool in endocrine practice. It should always be done in conjunction with clinical assessment of the patient, including complaints, history of associated diseases, and concomitant medications as well as careful physical examination. Any discrepancy between clinical and laboratory data deserves careful attention. Blind reliance on laboratory reports often leads to erroneous diagnosis and treatment. Laboratory personnel have neither contact with the patient nor can they access the patient’s history, or drug therapy. Thus, it is the responsibility of the treating physician, especially the endocrine consult, to synthesize the entire information necessary for proper interpretation of the hormonal tests.

Unfortunately, many endocrine training programs do not include actual exposure of the trainees to the intricacies of laboratory diagnosis and do not require competency in the interpretation of hormonal tests. This needs to become a required part of training programs. This review is only a partial introduction to the most basic and commonly encountered potential pitfalls in laboratory practice. We believe that active involvement of clinical pathology mentors in the comprehensive training of future endocrinologists is essential.

## Data Availability

Not applicable.

## Abbreviations

ACTH: Adrenocorticotropic Hormone
B-HCG: Beta Human Chorionic Gonadotropin bHCG
CAH: Congenital Adrenal Hyperplasia
CBG: Cortisol Binding Globulin
CYP3A4: Cytochrome P450 3A4
DST: Dexamethasone Suppression Test
FDA: United States Food and Drug Administration
GFC: Gel Filtration Chromatography
HAB: Heterophile Antibodies
HAMA: Human Anti-Mouse Antibodies
HPA: Hypothalamic-Pituitary-Adrenal
IFMA: Immunofluorometric Assay
ILMA: Immunoluminometric Assay
IRMA: Immunoradiometric Assay
LC-MS/MS: Liquid Chromatography-tandem Mass Spectrometry
PEG: Polyethylene Glycol
RIA: Radioimmunoassay
SHBG: Sex Hormone Binding Globulin
SLE: Systemic Lupus Erythematosus
T3: Triiodothyronine
T4: Thyroxine
TBG: Thyroxine Binding Globulin
TSH: Thyroid Stimulating Hormone
UFC: Urinary Free Cortisol
